# An Analytical Model for Squeeze-Film Damping of Perforated Torsional Microplates Resonators

**DOI:** 10.3390/s150407388

**Published:** 2015-03-25

**Authors:** Pu Li, Yuming Fang

**Affiliations:** 1 School of Mechanical Engineering, Southeast University, Jiangning, Nanjing 211189, China; 2 Nanjing University of Posts and Telecommunications, Nanjing 210003, China; E-Mail: fangym@njupt.edu.cn

**Keywords:** perforated torsion microplate, squeeze-film damping, quality factor

## Abstract

Squeeze-film damping plays a significant role in the performance of micro-resonators because it determines their quality factors. Perforations in microstructures are often used to control the squeeze-film damping in micro-resonators. To model the perforation effects on the squeeze-film damping, many analytical models have been proposed, however, most of the previous models have been concerned with the squeeze-film damping due to the normal motion between the perforated vibrating plate and a fixed substrate, while there is a lack of works that model the squeeze-film damping of perforated torsion microplates, which are also widely used in MEMS devices. This paper presents an analytical model for the squeeze-film damping of perforated torsion microplates. The derivation in this paper is based on a modified Reynolds equation that includes compressibility and rarefaction effects. The pressure distribution under the vibrating plate is obtained using the double sine series. Closed-form expressions for the stiffness and the damping coefficients of the squeeze-film are derived. The accuracy of the model is verified by comparing its results with the finite element method (FEM) results and the experimental results available in the literature. The regime of validity and limitations of the present model are assessed.

## 1. Introduction

Squeeze-film damping is one of the most dominant factors that limits the performance of high-Q micro-resonators. Perforations in microstructures play a significant role in controlling the squeeze-film damping. In the past, to model perforation effects on the squeeze-film damping in the micro-resonators, many methods have been proposed [1,2,3,4,5,6,7,8,9,10,11,12,13,14,15,16,17,18,19,20]. We can identify two groups in this respect. The first group [1,2,3,4,5,6,7] used the finite element method (FEM) to model the squeeze-film damping. The second group [8,9,10,11,12,13,14,15,16,17,18,19,20] is devoted to presenting an easy-to-use analytical model for calculating the squeeze-film damping. In principle, the FEM based numerical methods are the most accurate way to evaluate the squeeze-film damping in micro-resonators. However, a perforated microstructure might consist of thousands of tiny holes [18]. Direct simulation of such structures with 3-D FEM tools is time consuming and non-transparent [7,18]. In fact, many micro-resonators have a simple structure and simple boundary conditions. For these simple structures with simple boundary conditions, the analytical models can provide a better insight into the physical characteristic of devices. The aim of this paper is to provide an analytical model for estimating the squeeze-film damping in a perforated torsion microplate. Next, we summarize the analytical models [8,9,10,11,12,13,14,15,16,17,18,19,20] for the squeeze-film damping of perforated microplates.

Two approaches are used in the published analytical models. In the first approach, the vibration microplate is considered as a set of uniformly distributed cells. Each cell contains a single hole. The pressure profile is repetitive. The Reynolds equation is solved within the single cell using suitable boundary conditions. The total damping is calculated by multiplying the damping due to the single cell by the total number of cells. This first approach was followed by Skvor [8], Bao *et al.* [9], Mohite *et al.* [10,11], Kowk *et al.* [12], Homentcovschi and Miles [13,14], Homentcovschi *et al.* [15]. In 1967, Skovr [8] first derived an analytical expression using the first approach to determine the squeeze-film damping of a perforated microplate, however, the derivation didn’t consider the air compressibility, the edge effect and the flow resistance of the holes. In 2002, Bao *et al.* [9] extended the Skvor model by including the flow resistance of the holes. In 2004, Homentcovschi and Miles [13] also used the Skvor model and included the resistance of the perforations. The optimum number of holes was determined. In 2005 and 2008, Mohite *et al.* [10,11] extended Skvor’s model to include compressibility, rarefaction and inertia effects. In 2005, Kowk *et al.* [12] also derived a new model to include perforations, however, Kowk’s model is only valid for large perforations. In 2007, Homentcovschi and Miles [14] extended the Skvor model by including the edge correction. In 2010, Homentcovschi *et al.* [15] presented a new model for a perforated microplate outside the lubrication approximation.

The second approach is to modify the Reynolds equation by adding new terms and coefficients to account for the influence of the holes on the pressure of the flow through the microstructure. The modified Reynolds equation is then solved within the whole plate to directly get the overall damping. This second approach was followed by Veijola and Mattila [16], Bao *et al.* [17], Veijola [18], Pandey *et al.* [19] and Li *et al.* [20]. In 2001, Veijola and Mattila [16] were the first to derive an extended Reynolds equation that has an additional “leakage” term due to the perforations. In 2003, by adding a penetrating term to the fluid continuity equation, Bao *et al.* [17] derived a modified Reynolds equation under the assumption of incompressible flow. In 2006, Veijola [18] modeled the perforation effect by calculating the equivalent electrical impedance for squeeze film damping, the flow resistance of the holes, end effect of the holes and the compressibility effect. In 2007, Pandey *et al.* [19] extended Bao’s model to include the compressibility effect and rarefaction effect. The previous models based on the second approach are only suitable to deal with rectangular perforated microplates, but circular perforated microplates are also widely used in micro-resonators. In 2014, we [20] presented an analytical model for calculating the squeeze-film damping in perforated circular microplates. Our previous work [20] is also based on the second approach.

Torsion perforated microplates are also common elements in micro-resonators. However, there are few works on analytical modeling of the squeeze-film damping of torsion perforated microplates. All the previous works focus on perforated microplates vibrating in the direction normal to the substrate. This paper presents an analytical model for calculating the squeeze-film damping in a perforated torsion microplate. This paper is based on the second approach. Table 1 lists the contributions of the previous models and the proposed model based on the second approach. The outline of this paper is as follows: two types of torsion plates are often used in micro-resonators, so Section 2 and Section 3 present two analytical models for calculating the squeeze-film damping in the two types of torsion plate, respectively. The pressure in the air gap is represented by the double sine series. Analytical expressions for the squeeze-film damping and spring constants have been found. Section 4 calculates the squeeze-film damping using the present models, and compares the calculated results with the FEM results and the experimental data [21]. The regime of validity and limitations of the present models are assessed. Finally, a conclusion is given in Section 5.

**Table 1:** Comparison of the previous models and the proposed model based on the second approach.

Publication Year	Reference	Contributions and Structure Assumed in Model
2001	Veijola and Mattila [16]	An extended Reynolds equation is first derived by adding an additional “leakage” term due to the perforations. Rigid rectangular microplate is considered. The microplate is operated in normal direction to the substrate.
2003	Bao *et al.* [17]	A modified Reynolds equation under the assumption of incompressible flow is derived by adding a penetrating term to fluid continuity equation. Rigid rectangular microplate is considered. The microplate is operated in normal direction to the substrate.
2006	Veijola [18]	The perforation effect is model by calculating the equivalent electrical impedance for squeeze film damping, the flow resistance of the holes, end effect of the holes and the compressibility effect. Rigid rectangular microplate is considered. The microplate is operated in normal direction to the substrate.
2007	Pandey *et al.* [19]	Bao’s model [17] was extended to include the compressibility effect and rarefaction effect. Rigid and flexible rectangular microplates are considered. The microplate is operated in normal direction to the substrate.
2014	Li *et al.* [20]	The modified Reynolds equation presented by Pandey *et al.* [19] was extended to model the rigid and flexible circular microplates. The microplate is operated in normal direction to the substrate.
	Proposed model	The modified Reynolds equation presented by Pandey *et al.* [19] was extended to model the torsion microplate.

## 2. Analytical Modeling of Squeeze-Film Damping for the First Type Resonator

Current structures in torsion micro-resonators can be classified into two main types. In the first type, the torsion plate is symmetric with respect to the rotation axis. In the second type, the torsion plate is asymmetric. In this section, we focus on the squeeze-film damping in the first type of the torsion micro-resonators.

### 2.1. Governing Equations

**Figure 1:**
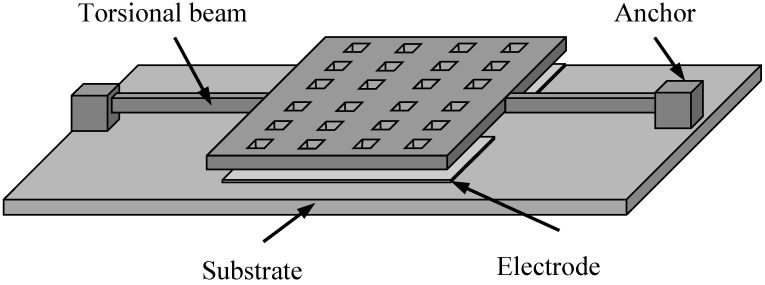
A schematic drawing of a perforated torsion microplate supported by two torsion beams.

Figure 1 shows a schematic drawing of a torsional micro-resonator. The device is a rigid rectangular oscillating microplate under the effect of squeeze-film damping. The rectangular microplate is uniformly perforated with square holes. The rectangular microplate is supported by two torsion beams which in turn are mounted on two anchors fixed to the rigid substrate. The two torsion beams can be treated as two torsion springs. The rectangular microplate is symmetric with respect to the rotation axis. For convenience, we will refer to this as a type I device. The perforated rectangular plate is excited by an electrostatic force. There are two electrodes in the substrate. $g_{0}$ is the zero-voltage air gap spacing. The thickness of the two electrodes can be neglected.

**Figure 2:**
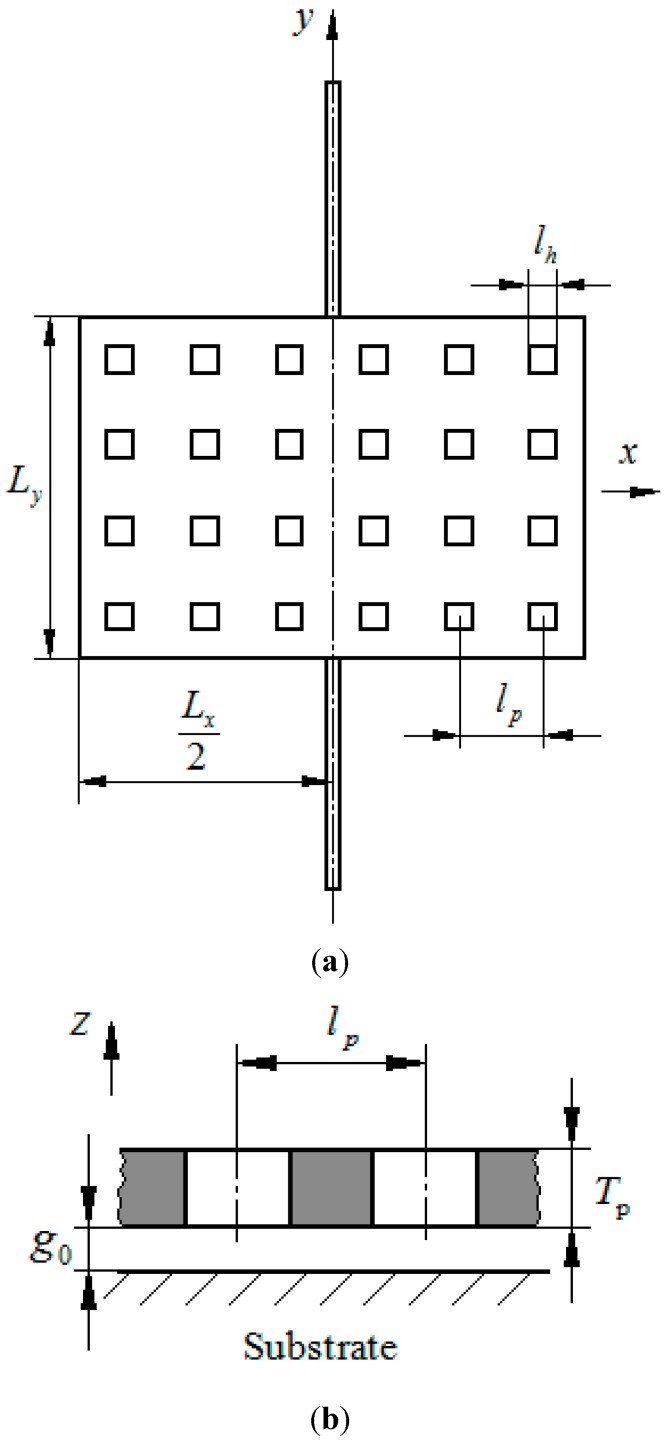
A schematic drawing of a perforated torsion microplate (Type I). (**a**) Top view of the perforated microplate with uniformly distributed holes; (**b**) cross-sectional view.

Figure 2 shows a top view and a cross-sectional view of the rectangular microplate. The plate has *N_x_* × *N_y_* uniformly distributed square holes of size *l_h_* with a pitch *l_p_* along both the *x* and *y*-directions. Here, *N_x_* is the number of holes along *x*-direction and *N_y_* is the number of holes along *y*-directions. *L_x_*, *L_y_* and *T_p_* are the length, width and thickness of the plate, respectively.

In 2002, Bao *et al.* [9] first presented a modified Reynolds equation for the rectangular perforated microplate vibrating in the direction normal to the substrate by subtracting the pressure relief due to the perforations. However, the compressibility effect and the rarefaction effect of the air in the gap are not included in their work. In 2007, Pandey *et al.* [19] extended Bao’s model to include the compressibility effect and rarefaction effect.

The modified Reynolds equation presented by Pandey *et al.* [19] under isothermal conditions is:

(1) $$\left\{\frac{\partial }{\partial x}\left(p\frac{Q_{\text{ch}}h^{3}}{12\text{μ}}\frac{\partial p}{\partial x}\right)+\frac{\partial }{\partial y}\left(p\frac{Q_{\text{ch}}h^{3}}{12\text{μ}}\frac{\partial p}{\partial y}\right)\right\}-\frac{Q_{\text{th}}\beta^{2}b^{2}}{8\text{μ}T_{\text{eff}}\text{η}(\text{β})}p\left(p-p_{a}\right)=\frac{\partial \left(ph\right)}{\partial t}$$

where h(t) is the squeeze-film thickness, p(x,y,t) is the pressure of air in the gap, *p_a_* is the ambient pressure, μ is the viscosity, $\text{β}=\frac{b}{a}$, $b=\frac{1.096l_{h}}{2}$ is the equivalent hole radius [11], $a=\frac{l_{p}}{\sqrt{\text{π}}}$ is the area equivalent outer radius of the pressure cell [11], $T_{\text{eff}}=T_{\text{P}}+(3\text{π}b/8)$ is the effective hole length which includes the hole length *T_p_* and an equivalent length to account for the end effect of the hole, $\text{η}(\text{β})=1+\frac{3b^{4}K(\text{β})Q_{\text{th}}}{\text{16}T_{\text{eff}}g_{0}^{3}Q_{\text{ch}}}$ and $K(\text{β})=4{\text{β}}^{2}-{\text{β}}^{4}-4\ln\text{β}-3$, *Q*_ch_ and *Q*_th_ are the flow rate factors which account for the rarefaction effect in the flow through the parallel plates and through the holes, respectively. The expressions for *Q*_ch_ and *Q*_th_ are:

(2) $$Q_{\text{ch}}=1+3\frac{0.01807\sqrt{\text{π}}}{D_{0}}+6\frac{1.35355}{D_{0}^{1.17468}}$$

(3) $$Q_{\text{th}}=1+4Kn_{\text{th}}$$

where $D_{0}=\frac{\sqrt{\text{π}}}{2Kn_{\text{ch}}}$, $Kn_{\text{ch}}=\frac{\text{λ}}{g_{0}}$, $Kn_{\text{th}}=\frac{\text{λ}}{b}$ and $\text{λ}=\frac{0.0068}{p_{a}}$ at ambient temperature and pressure *p_a_*.

The previous works presented by Bao *et al.* [17] and Pandey *et al.* [19] have dealt with the squeeze-film damping due to the normal motion between two parallel surfaces. The squeeze-film thickness in their works is $h(t)=g_{0}+A_{0}{\text{e}}^{\text{j}\omega t}$, where $A_{0}{\text{e}}^{\text{j}\omega t}$ is the displacement of the moving plate. In this section, for the torsion plate, the squeeze-film thickness can be expressed as:

(4) $$h(t)=g_{0}+x\cdot \theta_{0}{\text{e}}^{\text{j}\omega t}\begin{array}{cc} , & -\frac{L_{x}}{2}\leq x\leq \frac{L_{x}}{2} \end{array}$$

where $\theta_{0}{\text{e}}^{\text{j}\omega t}$ is the angular displacement of the rotating plate.

Equation (1) can be linearized under the assumption of small amplitude vibration $\left(g_{0}>>x\cdot \theta_{0}\right)$ and small pressure variation ($p_{a}>>\Delta p$), where $p=p_{a}+\Delta p$. For convenience, we introduce the follow nondimensional variables:

(5) $$P(x,y,t)=\frac{\text{Δ}p}{p_{a}}\;H(t)=h_{0}{\text{e}}^{\text{j}\omega t}$$

where $h_{0}=\frac{\theta_{0}}{g_{0}}$. Substituting Equation (5) into Equation (1), and linearing the outcome around *p_a_* and $g_{0}$, leads to:

(6) $$\frac{\partial^{2}P}{\partial x^{2}}+\frac{\partial^{2}P}{\partial y^{2}}-\frac{P}{L^{2}}=\alpha^{2}\cdot \left(\frac{\partial P}{\partial t}+x\frac{\partial H}{\partial t}\right)$$

where $L=\sqrt{\frac{2g_{0}^{3}T_{\text{eff}}\text{η}(\text{β})Q_{\text{ch}}}{3{\text{β}}^{2}b^{2}Q_{\text{th}}}}$ is the characteristic length and ${\text{α}}^{2}=\frac{12\text{μ}}{Q_{\text{ch}}p_{a}g_{0}^{2}}$. The boundary conditions for the torsion plate are:

(7) $$P(-\frac{L_{x}}{2},y,t)=P(+\frac{L_{x}}{2},y,t)=P(x,-\frac{L_{y}}{2},t)=P(x,+\frac{L_{y}}{2},t)=0$$

### 2.2. Analytical Model for the Type I Device

The solution of Equation (6) can be approximated by the following form:

(8) $$P(x,y,t)=\sum_{m=1}^{\infty }\sum_{n=1}^{\infty }a_{mn}\text{sin}\left[\frac{m\text{π}}{L_{x}}(x+\frac{L_{x}}{2})\right]\cdot \text{sin}\left[\frac{n\text{π}}{L_{y}}(y+\frac{L_{y}}{2})\right]\cdot {\text{e}}^{\text{j}\omega t}$$

where $a_{mn}$ is the complex amplitude to be determined. Obviously, Equation (8) satisfies the boundary condition (Equation (7)). Substituting Equation (8) and $H(t)=h_{0}{\text{e}}^{\text{j}\omega t}$ into Equation (6), leads to:

(9) $$\sum_{m=1}^{\infty }\sum_{n=1}^{\infty }a_{mn}\left\{-{\left(\frac{m\text{π}}{L_{x}}\right)}^{\text{2}}-{\left(\frac{n\text{π}}{L_{y}}\right)}^{\text{2}}-\frac{1}{L^{2}}-{\text{jωα}}^{2}\right\}\cdot \text{sin}\left[\frac{m\text{π}}{L_{x}}(x+\frac{L_{x}}{2})\right]\cdot \text{sin}\left[\frac{n\text{π}}{L_{y}}(y+\frac{L_{y}}{2})\right]=\text{jω}\cdot {\text{α}}^{2}xh_{0}$$

Multiplying both sides of Equation (9) by $\text{sin}\left[\frac{m\text{π}}{L_{x}}(x+\frac{L_{x}}{2})\right]\cdot \text{sin}\left[\frac{n\text{π}}{L_{y}}(y+\frac{L_{y}}{2})\right]$, and integrating the results from $x=-\frac{L_{x}}{2}$ to $\frac{L_{x}}{2}$ and $y=-\frac{L_{y}}{2}$ to $\frac{L_{y}}{2}$ leads to:

(10) $$a_{mn}=\{\begin{array}{l} \begin{array}{cc} {\hat{a}}_{mn}^{\text{R}}+\text{j}\cdot {\hat{a}}_{mn}^{\text{I}}=\left(\frac{8L_{x}}{mn{\text{π}}^{2}}\right)\frac{\text{jω}\cdot {\text{α}}^{2}h_{0}}{\left\{{\left(\frac{m\text{π}}{L_{x}}\right)}^{\text{2}}+{\left(\frac{n\text{π}}{L_{y}}\right)}^{\text{2}}+\frac{1}{L^{2}}+{\text{jωα}}^{2}\right\}} & \begin{array}{cc} \text{for} & m=2,4,6;n=1,3,5 \end{array} \end{array}\\ \begin{array}{cc} 0 & , \end{array}\text{otherwise}. \end{array}$$

where ${\hat{a}}_{mn}^{\text{R}}$ and ${\hat{a}}_{mn}^{\text{I}}$ are the real and imaginary parts of $a_{mn}$, the expressions of ${\hat{a}}_{mn}^{\text{R}}$ and ${\hat{a}}_{mn}^{\text{I}}$ are as follows:

(11) $${\hat{a}}_{mn}^{\text{R}}=h_{0}\left(\frac{8L_{x}}{mn{\text{π}}^{2}}\right)\frac{\omega^{2}\alpha^{4}}{\left\{{\left[{\left(\frac{m\text{π}}{L_{x}}\right)}^{\text{2}}+{\left(\frac{n\text{π}}{L_{y}}\right)}^{\text{2}}+\frac{1}{L^{2}}\right]}^{2}+\omega^{2}\alpha^{4}\right\}}$$

(12) $${\hat{a}}_{mn}^{\text{I}}=h_{0}\left(\frac{8L_{x}}{mn{\text{π}}^{2}}\right)\frac{{\text{ωα}}^{2}\left[{\left(\frac{m\text{π}}{L_{x}}\right)}^{\text{2}}+{\left(\frac{n\text{π}}{L_{y}}\right)}^{\text{2}}+\frac{1}{L^{2}}\right]}{\left\{{\left[{\left(\frac{m\text{π}}{L_{x}}\right)}^{\text{2}}+{\left(\frac{n\text{π}}{L_{y}}\right)}^{\text{2}}+\frac{1}{L^{2}}\right]}^{2}+{\text{ω}}^{2}{\text{α}}^{4}\right\}}$$

The equation of motion for the torsion plate shown in Figure 1 can be expressed as:

(13) $$\hat{J}\ddot{\theta }+{\hat{k}}_{\theta }\theta =V(t)+T_{\text{Squeeze}}$$

where $\hat{J}$ is the moment of inertia about the rotation axis, ${\hat{k}}_{\theta }$ is the torsional stiffness, V(t) is a harmonic excitation, $T_{\text{Squeeze}}$ is the total torque acting on the plate owing to the pressure of the squeeze gas film. $T_{\text{Squeeze}}$ can be calculated by integrating the pressure distribution on the surface of the plate:

(14) $$T_{\text{Squeeze}}=\int_{-\frac{L_{y}}{2}}^{\frac{L_{y}}{2}}\int_{-\frac{L_{x}}{2}}^{\frac{L_{x}}{2}}\left(p-p_{a}\right)\cdot x\text{d}x\text{d}y=\int_{-\frac{L_{y}}{2}}^{\frac{L_{y}}{2}}\int_{-\frac{L_{x}}{2}}^{\frac{L_{x}}{2}}\Delta p\cdot x\text{d}x\text{d}y$$

so the total damping torque and the total spring torque acting on the plate due to the squeeze-film are:

(15) $$\begin{array}{l} T_{\text{damping}}=T_{\text{Squeeze}}^{\text{I}}=p_{a}\cdot \int_{-\frac{L_{y}}{2}}^{\frac{L_{y}}{2}}\int_{-\frac{L_{x}}{2}}^{\frac{L_{x}}{2}}\sum_{m=2,4,6\cdots }^{\infty }\sum_{n=1,3,5}^{\infty }{\hat{a}}_{mn}^{\text{I}}\text{sin}\left[\frac{m\text{π}}{L_{x}}(x+\frac{L_{x}}{2})\right]\cdot \text{sin}\left[\frac{n\text{π}}{L_{y}}(y+\frac{L_{y}}{2})\right]x\text{d}x\text{d}y\\ =-p_{a}h_{0}\cdot \sum_{m=2,4,6\cdots }^{\infty }\sum_{n=1,3,5}^{\infty }\left(\frac{16L_{x}^{3}L_{y}}{m^{\text{2}}n^{\text{2}}{\text{π}}^{4}}\right)\frac{\text{ω}\alpha^{2}\left[{\left(\frac{m\text{π}}{L_{x}}\right)}^{\text{2}}+{\left(\frac{n\text{π}}{L_{y}}\right)}^{\text{2}}+\frac{1}{L^{2}}\right]}{\left\{{\left[{\left(\frac{m\text{π}}{L_{x}}\right)}^{\text{2}}+{\left(\frac{n\text{π}}{L_{y}}\right)}^{\text{2}}+\frac{1}{L^{2}}\right]}^{2}+{\text{ω}}^{2}{\text{α}}^{4}\right\}} \end{array}$$

(16) $$\begin{array}{l} T_{\text{spring}}=T_{\text{Squeeze}}^{\text{R}}=p_{a}\cdot \int_{-\frac{L_{y}}{2}}^{\frac{L_{y}}{2}}\int_{-\frac{L_{x}}{2}}^{\frac{L_{x}}{2}}\sum_{m=2,4,6\cdots }^{\infty }\sum_{n=1,3,5}^{\infty }{\hat{a}}_{mn}^{\text{R}}\text{sin}\left[\frac{m\text{π}}{L_{x}}(x+\frac{L_{x}}{2})\right]\cdot \text{sin}\left[\frac{n\text{π}}{L_{y}}(y+\frac{L_{y}}{2})\right]x\text{d}x\text{d}y\\ =-p_{a}h_{0}\sum_{m=2,4,6\cdots }^{\infty }\sum_{n=1,3,5}^{\infty }\left(\frac{16L_{x}^{3}L_{y}}{m^{\text{2}}n^{\text{2}}{\text{π}}^{4}}\right)\frac{{\text{ω}}^{2}{\text{α}}^{4}}{\left\{{\left[{\left(\frac{m\text{π}}{L_{x}}\right)}^{\text{2}}+{\left(\frac{n\text{π}}{L_{y}}\right)}^{\text{2}}+\frac{1}{L^{2}}\right]}^{2}+{\text{ω}}^{2}{\text{α}}^{4}\right\}} \end{array}$$

where $T_{\text{Squeeze}}^{\text{R}}$ and $T_{\text{Squeeze}}^{\text{I}}$ are the real and imaginary parts of $T_{\text{Squeeze}}$. The corresponding damping constant $C_{\theta }^{\text{I}}$ and the spring constant $K_{\theta }^{\text{I}}$ owing to the pressure of the squeeze gas film are given by:

(17) $$\begin{array}{l} C_{\theta }^{\text{I}}=\frac{-T_{\text{damping}}}{\theta_{0}\text{ω}}=\frac{p_{a}}{g_{0}}\cdot \sum_{m=2,4,6\cdots }^{\infty }\sum_{n=1,3,5}^{\infty }\left(\frac{16L_{x}^{3}L_{y}}{m^{\text{2}}n^{\text{2}}{\text{π}}^{4}}\right)\frac{{\text{α}}^{2}\left[{\left(\frac{m\text{π}}{L_{x}}\right)}^{\text{2}}+{\left(\frac{n\text{π}}{L_{y}}\right)}^{\text{2}}+\frac{1}{L^{2}}\right]}{\left\{{\left[{\left(\frac{m\text{π}}{L_{x}}\right)}^{\text{2}}+{\left(\frac{n\text{π}}{L_{y}}\right)}^{\text{2}}+\frac{1}{L^{2}}\right]}^{2}+{\text{ω}}^{2}{\text{α}}^{4}\right\}}\\ =\frac{p_{a}}{g_{0}}\cdot \frac{\text{σ}}{\text{ω}}\cdot \sum_{m=2,4,6\cdots }^{\infty }\sum_{n=1,3,5}^{\infty }\left(\frac{16L_{x}^{3}L_{y}}{m^{\text{2}}n^{\text{2}}{\text{π}}^{4}}\right)\frac{\left[{\left(m\text{π}\right)}^{\text{2}}+{\left(n\text{π}\frac{L_{x}}{L_{y}}\right)}^{\text{2}}+\frac{L_{x}^{2}}{L^{2}}\right]}{\left\{{\left[{\left(m\text{π}\right)}^{\text{2}}+{\left(n\text{π}\frac{L_{x}}{L_{y}}\right)}^{\text{2}}+\frac{L_{x}^{2}}{L^{2}}\right]}^{2}+{\text{σ}}^{2}\right\}} \end{array}$$

(18) $$\begin{array}{l} K_{\theta }^{\text{I}}=\frac{-T_{\text{spring}}}{{\text{θ}}_{0}}=\frac{p_{a}}{g_{0}}\cdot \sum_{m=2,4,6\cdots }^{\infty }\sum_{n=1,3,5}^{\infty }\left(\frac{16L_{x}^{3}L_{y}}{m^{\text{2}}n^{\text{2}}{\text{π}}^{4}}\right)\frac{{\text{ω}}^{2}{\text{α}}^{4}}{\left\{{\left[{\left(\frac{m\text{π}}{L_{x}}\right)}^{\text{2}}+{\left(\frac{n\text{π}}{L_{y}}\right)}^{\text{2}}+\frac{1}{L^{2}}\right]}^{2}+{\text{ω}}^{2}{\text{α}}^{4}\right\}}\\ =\frac{p_{a}}{g_{0}}\cdot \sum_{m=2,4,6\cdots }^{\infty }\sum_{n=1,3,5}^{\infty }\left(\frac{16L_{x}^{3}L_{y}}{m^{\text{2}}n^{\text{2}}{\text{π}}^{4}}\right)\frac{{\text{σ}}^{2}}{\left\{{\left[{\left(m\text{π}\right)}^{\text{2}}+{\left(n\text{π}\frac{L_{x}}{L_{y}}\right)}^{\text{2}}+\frac{L_{x}^{2}}{L^{2}}\right]}^{2}+{\text{σ}}^{2}\right\}} \end{array}$$

where $\text{σ}={\text{ωα}}^{2}L_{x}^{2}=\frac{12\text{μω}L_{x}^{2}}{Q_{\text{ch}}p_{a}g_{0}^{2}}$ is the squeeze number.

In MEMS area, torsion microresonators are usually operated at low frequency (the first natural frequency). In this case, the squeeze number is very small; thus $K_{\theta }^{\text{I}}\approx 0$. The air flow could be treated as “incompressible air”. Equation (17) then reduces to:

(19) $$C_{\theta }^{\text{I}}=\frac{p_{a}}{g_{0}}\cdot \frac{\text{σ}}{\text{ω}}\cdot \sum_{m=2,4,6\cdots }^{\infty }\sum_{n=1,3,5}^{\infty }\left(\frac{16L_{x}^{3}L_{y}}{m^{\text{2}}n^{\text{2}}{\text{π}}^{4}}\right)\cdot {\left[{\left(m\text{π}\right)}^{\text{2}}+{\left(n\text{π}\frac{L_{x}}{L_{y}}\right)}^{\text{2}}+\frac{L_{x}^{2}}{L^{2}}\right]}^{-1}$$

## 3. Analytical Modeling of Squeeze-Film Damping for the Second Type Resonator

**Figure 3:**
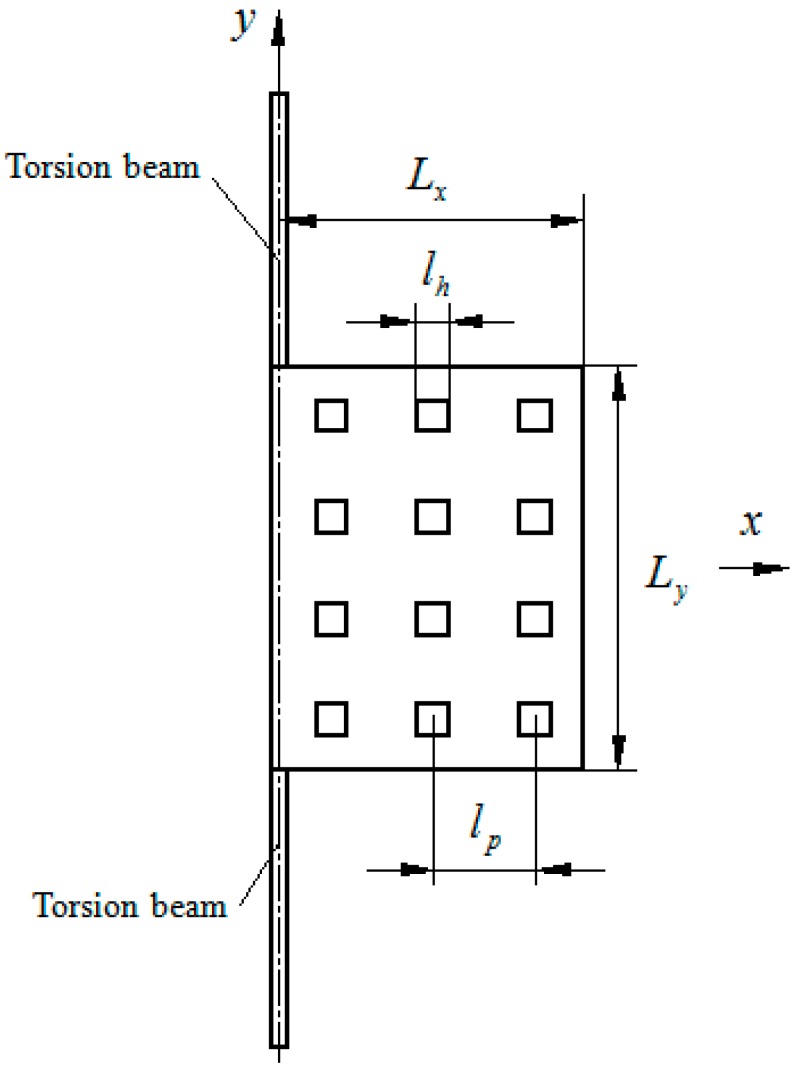
Top view of the perforated torsion microplate (type II).

The torsion plate in the second type resonator is asymmetric with respect to the rotation axis. Figure 3 shows the top view of the torsion plate:

For convenience, we will refer to this as the type II device. A similar analysis as the one given for the squeeze-film damping in the type I device can be given for the type II device. In the type II device, the squeeze-film thickness can be expressed as:

(20) $$h(t)=g_{0}+x\cdot {\text{θ}}_{0}{\text{e}}^{\text{jω}t}\begin{array}{cc} , & 0\leq x\leq L_{x} \end{array}$$

where ${\text{θ}}_{0}{\text{e}}^{\text{j}\omega t}$ is the angular displacement of the rotating plate. The boundary conditions for the torsion plate are:

(21) $$P(0,y,t)=P(+L_{x},y,t)=P(x,-\frac{L_{y}}{2},t)=P(x,+\frac{L_{y}}{2},t)=0$$

For the torsion plate, we can choose the following function to approximate the pressure in the air gap:

(22) $$P(x,y,t)=\sum_{m=1}^{\infty }\sum_{n=1}^{\infty }b_{mn}\text{sin}\left(\frac{m\text{π}}{L_{x}}x\right)\cdot \text{sin}\left[\frac{n\text{π}}{L_{y}}(y+\frac{L_{y}}{2})\right]\cdot {\text{e}}^{\text{j}\omega t}$$

where $b_{mn}$ is the complex amplitude to be determined. Obviously, Equation (22) satisfies the boundary conditions (Equation (21)).

Substituting Equation (22) and $H(t)=h_{0}{\text{e}}^{\text{j}\omega t}$ into Equation (6), leads to:

(23) $$\sum_{m=1}^{\infty }\sum_{n=1}^{\infty }b_{mn}\left\{-{\left(\frac{m\text{π}}{L_{x}}\right)}^{\text{2}}-{\left(\frac{n\text{π}}{L_{y}}\right)}^{\text{2}}-\frac{1}{L^{2}}-{\text{jωα}}^{2}\right\}\cdot \text{sin}\left(\frac{m\text{π}}{L_{x}}x\right)\cdot \text{sin}\left[\frac{n\text{π}}{L_{y}}(y+\frac{L_{y}}{2})\right]=\text{jω}\cdot {\text{α}}^{2}xh_{0}$$

Multiplying both sides of Equation (23) by $\text{sin}\left(\frac{m\text{π}}{L_{x}}x\right)\cdot \text{sin}\left[\frac{n\text{π}}{L_{y}}(y+\frac{L_{y}}{2})\right]$, and integrating the results from x=0 to $L_{x}$ and $y=-\frac{L_{y}}{2}$ to $\frac{L_{y}}{2}$ leads to:

(24) $$b_{mn}=\{\begin{array}{l} \begin{array}{cc} {\hat{b}}_{mn}^{\text{R}}+\text{j}\cdot {\hat{b}}_{mn}^{\text{I}}=\left(\frac{8L_{x}}{mn{\text{π}}^{2}}\right)\frac{{\left(-1\right)}^{m}\cdot \text{jω}\cdot {\text{α}}^{2}h_{0}}{{\left(\frac{m\text{π}}{L_{x}}\right)}^{\text{2}}+{\left(\frac{n\text{π}}{L_{y}}\right)}^{\text{2}}+\frac{1}{L^{2}}+{\text{jωα}}^{2}}\begin{array}{cc} & \text{for} \end{array} & m=1,2,3,4\cdots ;n=1,3,5\cdots \end{array}\\ 0\begin{array}{cc} , & \end{array}\text{otherwise}. \end{array}$$

where ${\hat{b}}_{mn}^{\text{R}}$ and ${\hat{b}}_{mn}^{\text{I}}$ are the real and imaginary parts of $b_{mn}$, the expressions of ${\hat{b}}_{mn}^{\text{R}}$ and ${\hat{b}}_{mn}^{\text{I}}$ are as follows:

(25) $${\hat{b}}_{mn}^{\text{R}}={\left(-1\right)}^{m}{\hat{a}}_{mn}^{\text{R}}={\left(-1\right)}^{m}h_{0}\left(\frac{8L_{x}}{mn{\text{π}}^{2}}\right)\frac{{\text{ω}}^{2}{\text{α}}^{4}}{\left\{{\left[{\left(\frac{m\text{π}}{L_{x}}\right)}^{\text{2}}+{\left(\frac{n\text{π}}{L_{y}}\right)}^{\text{2}}+\frac{1}{L^{2}}\right]}^{2}+{\text{ω}}^{2}{\text{α}}^{4}\right\}}$$

(26) $${\hat{b}}_{mn}^{\text{I}}={\left(-1\right)}^{m}{\hat{a}}_{mn}^{\text{I}}={\left(-1\right)}^{m}h_{0}\left(\frac{8L_{x}}{mn{\text{π}}^{2}}\right)\frac{{\text{ωα}}^{2}\left[{\left(\frac{m\text{π}}{L_{x}}\right)}^{\text{2}}+{\left(\frac{n\text{π}}{L_{y}}\right)}^{\text{2}}+\frac{1}{L^{2}}\right]}{\left\{{\left[{\left(\frac{m\text{π}}{L_{x}}\right)}^{\text{2}}+{\left(\frac{n\text{π}}{L_{y}}\right)}^{\text{2}}+\frac{1}{L^{2}}\right]}^{2}+{\text{ω}}^{2}{\text{α}}^{4}\right\}}$$

The torque acting on the plate, excreted by the pressure in the film, is:

(27) $${\hat{T}}_{\text{Squeeze}}=\int_{-\frac{L_{y}}{2}}^{\frac{L_{y}}{2}}\int_{0}^{L_{x}}\left(p-p_{a}\right)\cdot x\text{d}x\text{d}y=\int_{-\frac{L_{y}}{2}}^{\frac{L_{y}}{2}}\int_{0}^{L_{x}}\Delta p\cdot x\text{d}x\text{d}y$$

so the damping torque and the spring torque acting on the plate due to the squeeze-film are:

(28) $$\begin{array}{l} {\hat{T}}_{\text{damping}}={\hat{T}}_{\text{Squeeze}}^{\text{I}}=p_{a}\cdot \int_{-\frac{L_{y}}{2}}^{\frac{L_{y}}{2}}\int_{0}^{L_{x}}\sum_{m=1}^{\infty }\sum_{n=1}^{\infty }{\hat{b}}_{mn}^{\text{I}}\text{sin}\left(\frac{m\text{π}}{L_{x}}x\right)\cdot \text{sin}\left[\frac{n\text{π}}{L_{y}}(y+\frac{L_{y}}{2})\right]x\text{d}x\text{d}y\\ =-p_{a}h_{0}\cdot \sum_{m=1,2,3\cdots }^{\infty }\sum_{n=1,3,5\cdots }^{\infty }\left(\frac{16L_{x}^{3}L_{y}}{m^{\text{2}}n^{\text{2}}{\text{π}}^{4}}\right)\frac{{\text{ωα}}^{2}\left[{\left(\frac{m\text{π}}{L_{x}}\right)}^{\text{2}}+{\left(\frac{n\text{π}}{L_{y}}\right)}^{\text{2}}+\frac{1}{L^{2}}\right]}{\left\{{\left[{\left(\frac{m\text{π}}{L_{x}}\right)}^{\text{2}}+{\left(\frac{n\text{π}}{L_{y}}\right)}^{\text{2}}+\frac{1}{L^{2}}\right]}^{2}+{\text{ω}}^{2}{\text{α}}^{4}\right\}} \end{array}$$

(29) $$\begin{array}{l} {\hat{T}}_{\text{spring}}={\hat{T}}_{\text{Squeeze}}^{\text{R}}=p_{a}\cdot \int_{-\frac{L_{y}}{2}}^{\frac{L_{y}}{2}}\int_{0}^{L_{x}}\sum_{m=1}^{\infty }\sum_{n=1}^{\infty }{\hat{b}}_{mn}^{\text{I}}\text{sin}\left(\frac{m\text{π}}{L_{x}}x\right)\cdot \text{sin}\left[\frac{n\text{π}}{L_{y}}(y+\frac{L_{y}}{2})\right]x\text{d}x\text{d}y\\ =-p_{a}h_{0}\cdot \sum_{m=1,2,3\cdots }^{\infty }\sum_{n=1,3,5\cdots }^{\infty }\left(\frac{16L_{x}^{3}L_{y}}{m^{\text{2}}n^{\text{2}}{\text{π}}^{4}}\right)\frac{{\text{ω}}^{2}{\text{α}}^{4}}{\left\{{\left[{\left(\frac{m\text{π}}{L_{x}}\right)}^{\text{2}}+{\left(\frac{n\text{π}}{L_{y}}\right)}^{\text{2}}+\frac{1}{L^{2}}\right]}^{2}+{\text{ω}}^{2}{\text{α}}^{4}\right\}} \end{array}$$

where ${\hat{T}}_{\text{Squeeze}}^{\text{R}}$ and ${\hat{T}}_{\text{Squeeze}}^{\text{I}}$ are the real and imaginary parts of ${\hat{T}}_{\text{Squeeze}}$. The corresponding damping constant $C_{\theta }^{\text{II}}$ and the spring constant $K_{\theta }^{\text{II}}$ owing to the pressure of the squeeze gas film are given by:

(30) $$\begin{array}{l} C_{\theta }^{\text{II}}=\frac{-{\hat{T}}_{\text{damping}}}{\theta_{0}\text{ω}}=\frac{p_{a}}{g_{0}}\cdot \sum_{m=1,2,3\cdots }^{\infty }\sum_{n=1,3,5\cdots }^{\infty }\left(\frac{16L_{x}^{3}L_{y}}{m^{\text{2}}n^{\text{2}}{\text{π}}^{4}}\right)\frac{{\text{α}}^{2}\left[{\left(\frac{m\text{π}}{L_{x}}\right)}^{\text{2}}+{\left(\frac{n\text{π}}{L_{y}}\right)}^{\text{2}}+\frac{1}{L^{2}}\right]}{\left\{{\left[{\left(\frac{m\text{π}}{L_{x}}\right)}^{\text{2}}+{\left(\frac{n\text{π}}{L_{y}}\right)}^{\text{2}}+\frac{1}{L^{2}}\right]}^{2}+{\text{ω}}^{2}{\text{α}}^{4}\right\}}\\ =\frac{p_{a}}{g_{0}}\cdot \frac{\text{σ}}{\text{ω}}\cdot \sum_{m=1,2,3\cdots }^{\infty }\sum_{n=1,3,5}^{\infty }\left(\frac{16L_{x}^{3}L_{y}}{m^{\text{2}}n^{\text{2}}{\text{π}}^{4}}\right)\frac{\left[{\left(m\text{π}\right)}^{\text{2}}+{\left(n\text{π}\frac{L_{x}}{L_{y}}\right)}^{\text{2}}+\frac{L_{x}^{2}}{L^{2}}\right]}{\left\{{\left[{\left(m\text{π}\right)}^{\text{2}}+{\left(n\text{π}\frac{L_{x}}{L_{y}}\right)}^{\text{2}}+\frac{L_{x}^{2}}{L^{2}}\right]}^{2}+\sigma^{2}\right\}} \end{array}$$

(31) $$\begin{array}{l} K_{\theta }^{\text{II}}=\frac{-{\hat{T}}_{\text{spring}}}{{\text{θ}}_{0}}=\frac{p_{a}}{g_{0}}\cdot \sum_{m=1,2,3\cdots }^{\infty }\sum_{n=1,3,5\cdots }^{\infty }\left(\frac{16L_{x}^{3}L_{y}}{m^{\text{2}}n^{\text{2}}{\text{π}}^{4}}\right)\frac{{\text{ω}}^{2}{\text{α}}^{4}}{\left\{{\left[{\left(\frac{m\text{π}}{L_{x}}\right)}^{\text{2}}+{\left(\frac{n\text{π}}{L_{y}}\right)}^{\text{2}}+\frac{1}{L^{2}}\right]}^{2}+{\text{ω}}^{2}{\text{α}}^{4}\right\}}\\ =\frac{p_{a}}{g_{0}}\cdot \sum_{m=1,2,3\cdots }^{\infty }\sum_{n=1,3,5}^{\infty }\left(\frac{16L_{x}^{3}L_{y}}{m^{\text{2}}n^{\text{2}}{\text{π}}^{4}}\right)\frac{{\text{σ}}^{2}}{\left\{{\left[{\left(m\text{π}\right)}^{\text{2}}+{\left(n\text{π}\frac{L_{x}}{L_{y}}\right)}^{\text{2}}+\frac{L_{x}^{2}}{L^{2}}\right]}^{2}+{\text{σ}}^{2}\right\}} \end{array}$$

where $\text{σ}={\text{ωα}}^{2}L_{x}^{2}=\frac{12\text{μω}L_{x}^{2}}{Q_{\text{ch}}p_{a}g_{0}^{2}}$ is the squeeze number. Table 2 lists the damping constants and spring constants for the type I and II microplates.

In the case of incompressible air, $K_{\theta }^{\text{II}}\approx 0$ and Equation (30) reduces to:

(32) $$C_{\theta }^{\text{II}}\approx \frac{p_{a}}{g_{0}}\cdot \frac{\text{σ}}{\text{ω}}\cdot \sum_{m=1,2,3\cdots }^{\infty }\sum_{n=1,3,5}^{\infty }\left(\frac{16L_{x}^{3}L_{y}}{m^{\text{2}}n^{\text{2}}{\text{π}}^{4}}\right)\cdot {\left[{\left(m\text{π}\right)}^{\text{2}}+{\left(n\text{π}\frac{L_{x}}{L_{y}}\right)}^{\text{2}}+\frac{L_{x}^{2}}{L^{2}}\right]}^{-1}$$

**Table 2:** The squeeze-film damping models for the type I and II microplates.

Devices	The Damping Constant		The Spring Constant
Type I	$C_{\theta }^{\text{I}}=\frac{p_{a}\text{σ}}{g_{0}\text{ω}}\cdot \sum_{m=2,4,6\cdots }^{\infty }\sum_{n=1,3,5}^{\infty }\frac{A_{mn}\left[B_{mn}+\frac{L_{x}^{2}}{L^{2}}\right]}{{\left[B_{mn}+\frac{L_{x}^{2}}{L^{2}}\right]}^{2}+{\text{σ}}^{2}}$	$K_{\theta }^{\text{I}}=\frac{p_{a}}{g_{0}}\cdot \sum_{m=2,4,6\cdots }^{\infty }\sum_{n=1,3,5}^{\infty }\frac{{\text{σ}}^{2}A_{mn}}{{\left[B_{mn}+\frac{L_{x}^{2}}{L^{2}}\right]}^{2}+{\text{σ}}^{2}}$	
Type II	$C_{\theta }^{\text{II}}=\frac{p_{a}\text{σ}}{g_{0}\text{ω}}\cdot \sum_{m=1,2,3\cdots }^{\infty }\sum_{n=1,3,5}^{\infty }\frac{A_{mn}\left[B_{mn}+\frac{L_{x}^{2}}{L^{2}}\right]}{{\left[B_{mn}+\frac{L_{x}^{2}}{L^{2}}\right]}^{2}+{\text{σ}}^{2}}$	$K_{\theta }^{\text{II}}=\frac{p_{a}}{g_{0}}\cdot \sum_{m=1,2,3\cdots }^{\infty }\sum_{n=1,3,5}^{\infty }\frac{{\text{σ}}^{2}A_{mn}}{{\left[B_{mn}+\frac{L_{x}^{2}}{L^{2}}\right]}^{2}+{\text{σ}}^{2}}$	
$A_{mn}=\left(\frac{16L_{x}^{3}L_{y}}{m^{\text{2}}n^{\text{2}}{\text{π}}^{4}}\right)$ and $B_{mn}=\left[{\left(m\text{π}\right)}^{\text{2}}+{\left(n\text{π}\frac{L_{x}}{L_{y}}\right)}^{\text{2}}\right]$			

## 4. Validation and Discussions

In this section, we first validate the present model by comparing its results with FEM (ANSYS) results at different perforation ratios. Pandey *et al.* [21] conducted experiments to measure the quality factors of a torsion micro-resonator at low pressure. The plate in their device is perforated. The experimental results obtained by Pandey *et al.* [21] are also used to verify the present model in this section.

### 4.1. Comparsions with the FEM Results for the Type I Torsion Microplate

**Figure 4:**
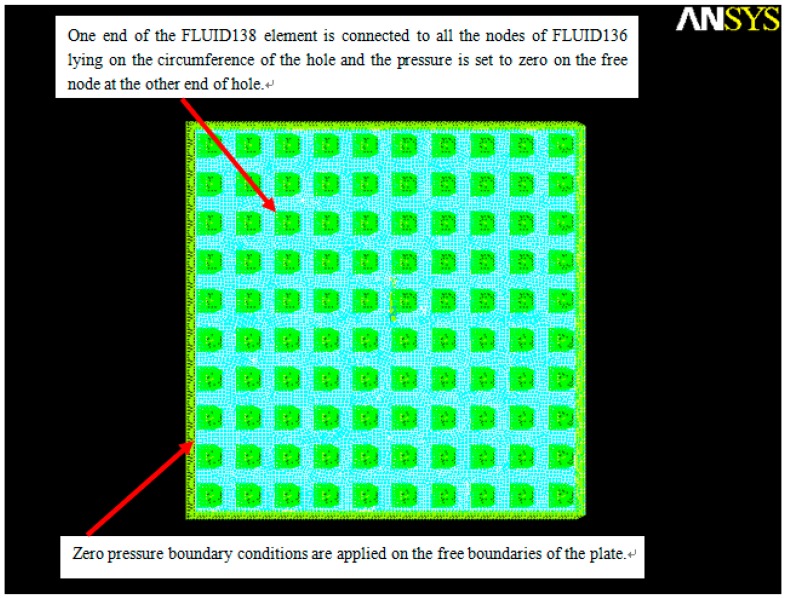
ANSYS model with boundary condition for the perforated micrplate (*l_h_*/*l_p_* = 0.4).

Now we compare the analytical results obtained by the present model with the FEM results at different perforation ratios (*l_h_*/*l_p_*). The torsion microplate with 100 holes (*N_x_* =*N_y_* = 10) shown in Figure 2 is considered in this subsection. The torsion plate is symmetric with respect to the rotation axis. The dimensions and parameters of the microplates used in simulations are listed in Table 3. The perforated torsion microplate is operated at 5000 Hz. The amplitude of the vibrating plate is very small ($A_{0}=\frac{g_{0}}{10L_{x}/2}$). In ANSYS, the four-noded FLUID136 element is used to model the viscous fluid flow behavior in the gap. The two-noded FLUID138 element is used to model the fluid flow through rectangular channels defined by holes. Zero pressure boundary conditions are applied on the free boundaries of the plate and the pressure is set to zero at the end of holes. Moving velocity condition in the *z*-direction is applied in the nodes. Figure 4 illustrates a typical ANSYS model with boundary conditions.

**Table 3:** Dimensions and parameters for the type I microplate.

Symbol	Description	Values	Unit
$L_{x}$	Length of the torsion microplate	500	µm
$L_{y}$	Widthth of the torsion microplate	500	µm
$T_{p}$	Thickness of the torsion microplate	10	µm
$N_{x}$	The total number of the holes along *x*-direction	10	
$N_{y}$	The total number of the holes along *y*-direction	10	
$l_{p}$	The pitch of the holes	50	µm
$g_{0}$	Gap spacing	5	µm
$p_{a}$	Ambient pressure	1.013 × 10^5^	N/m^2^
μ	Viscosity coefficient	1.83 × 10^−5^	N·s/m^2^

The infinite summations in the present model for squeeze-film damping were evaluated using MATLAB. Careful convergence studies were performed.

**Figure 5:**
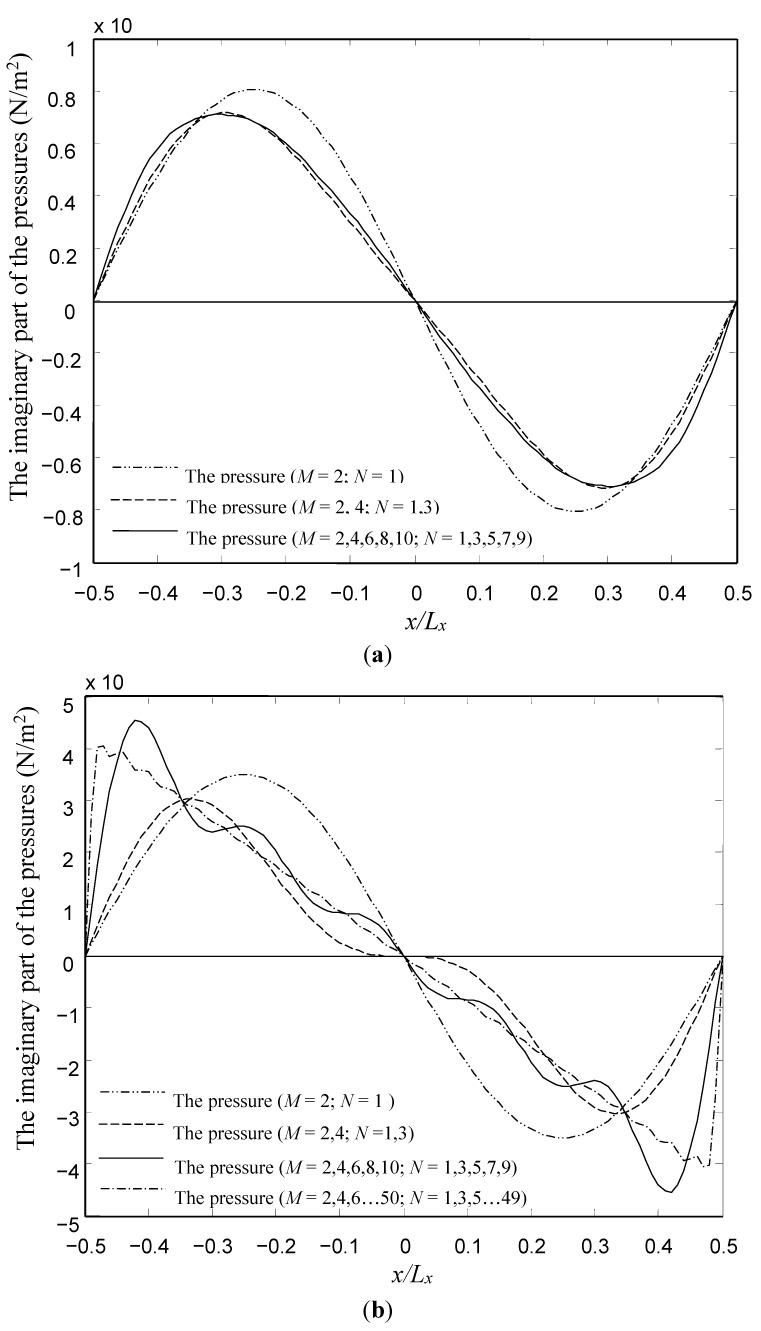
Convergence analysis of the series for the pressures in the air gap. (**a**) Comparison of the imaginary parts of the pressure at *y* = 0 in the case of *l_h_*/*l_p_* = 0.1 and (**b**) comparison of the imaginary parts of the pressure at *y* = 0 in the case of *l_h_*/*l_p_* = 0.8.

Figure 5a,b show the rate of convergence of the imaginary parts of the pressure at *y* = 0 for two microplates with *l_h_*/*l_p_* = 0.1 and 0.8, respectively. The results for the real parts of the pressure are not presented for the sake of brevity, and the tendency of the real parts of the pressure is similar to that for the imaginary parts. Obviously, for the microplate with small perforation ratio (*l_h_*/*l_p_* = 0.1), a total of 10 terms (*M* = 2, 4, 6, 8, 10 and *N* = 1, 3, 5, 7, 9) were sufficient to achieve convergence. The results for 10 terms are indistinguishable from the results for 20 terms (*M* = 2, 4, 6,…20 and *N* = 1, 3, 5…19). The discrepancy between the results for 10 terms and four terms (*M* = 2, 4 and *N* = 1, 3) is insignificant. However, for the microplate with large perforation ratio (*l_h_*/*l_p_* = 0.8), a total of 50 terms (*M* = 2, 4, 6,…50 and *N* = 1, 3, 5…49) were sufficient to achieve convergence. For this, and all other cases presented in this paper, a total of 50 terms were used to predict the squeeze-film damping.

**Figure 6:**
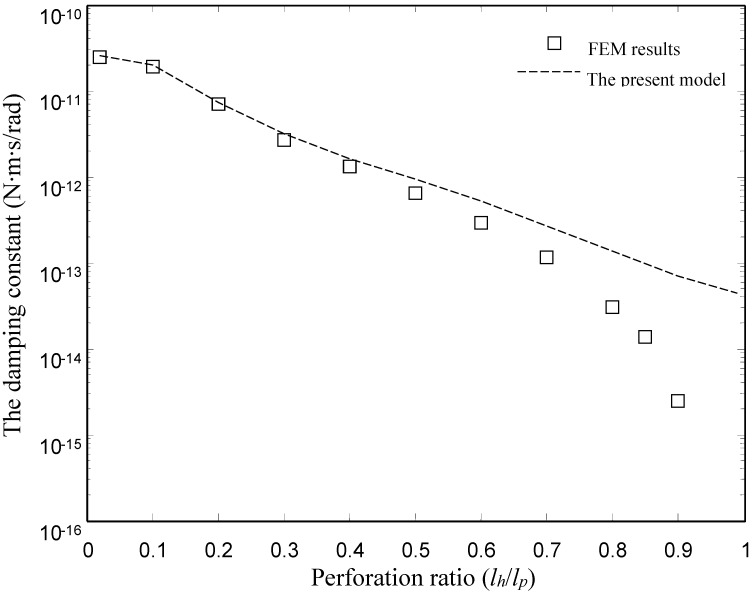
Comparison of the damping constant of the type I devices obtained by the FEM model and the present model.

**Figure 7:**
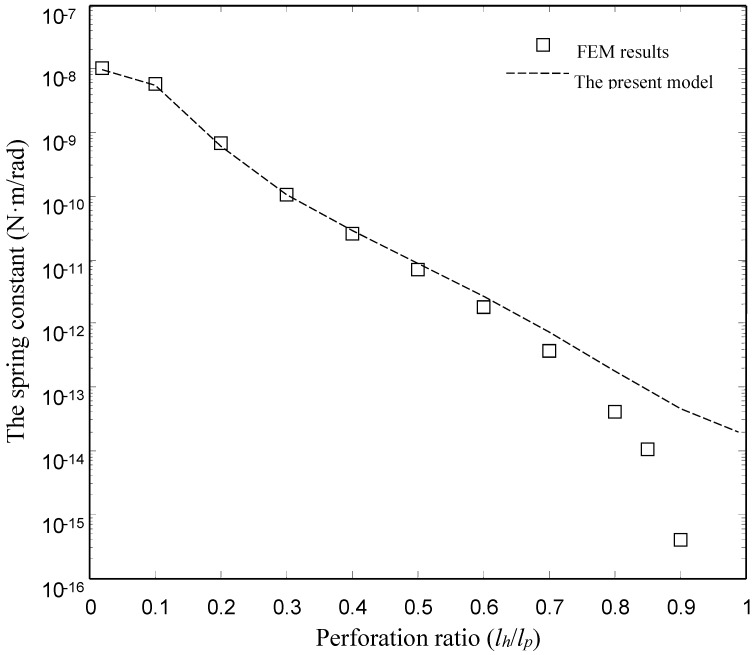
Comparison of the spring constant of the type I devices obtained by the FEM model and the present model.

Figure 6 and Figure 7 show the damping and spring constants of the microplates obtained using the FEM model and the present model for different values of perforation ratio (*l_h_*/*l_p_*). Table 4 lists the detailed values of the damping and spring constants obtained by the ANSYS model and the present model. For the FEM model, we varied perforation ratios from 0.02 to 0.9 for the same values of pitch (*l_p_* = 50 µm) and other dimensions as mentioned above. For the present model, we varied perforation ratios from 0.02 to 0.99. Obviously, the present model matches well with the numerical (FEM) results for the torsion plates with the smaller and medium perforation ratios. For the damping constants, in the case of smaller and medium perforation ratios (*l_h_*/*l_p_* ≤ 0.6), the present model gives values very close to the FEM results. For the spring constants, below *l_h_*/*l_p_* = 0.7, the present model gives values very close to the FEM results. Obviously, the discrepancy between the FEM model and the present model increases as the perforation ratio increases.

**Table 4:** The damping and spring constants obtained by the ANSYS model and the presnt model for the torsion microplate (Type I).

$\frac{l_{h}}{l_{p}}$	The Damping Constant		The Spring Constant	
	FEM Model	The Present Model	FEM Model	The Present Model
0.02	2.51 × 10^−11^	2.52 × 10^−11^	9.26 × 10^−9^	9.37 × 10^−9^
0.1	1.94 × 10^−11^	1.97 × 10^−11^	5.39 × 10^−9^	5.46 × 10^−9^
0.2	6.93 × 10^−12^	7.18 × 10^−12^	6.20 × 10^−10^	6.20 × 10^−10^
0.3	2.73 × 10^−12^	3.12 × 10^−12^	9.63 × 10^−11^	1.07 × 10^−10^
0.4	1.29 × 10^−12^	1.65 × 10^−12^	2.31 × 10^−11^	2.87 × 10^−11^
0.5	6.37 × 10^−13^	9.30 × 10^−13^	6.47 × 10^−12^	8.86 × 10^−12^
0.6	2.96 × 10^−13^	5.17 × 10^−13^	1.68 × 10^−12^	2.68 × 10^−12^
0.7	1.16 × 10^−13^	2.72 × 10^−13^	3.38 × 10^−13^	7.30 × 10^−13^
0.8	3.07 × 10^−14^	1.35 × 10^−13^	3.82 × 10^−14^	1.80 × 10^−13^
0.85	1.35 × 10^−14^	1.02 × 10^−13^	9.40 × 10^−15^	1.13 × 10^−13^
0.9	2.44 × 10^−15^	6.92 × 10^−14^	3.71 × 10^−16^	4.64 × 10^−14^
0.99	-	4.50 × 10^−14^	-	1.97 × 10^−14^

There is a clear discrepancy between the FEM model and the present model at large perforation ratios. Two reasons for the discrepancy are as follows:

First, the pressure function in the present model is assumed to be continuous. Obviously, the assumption is only reasonable for plates with smaller and medium perforation ratios. Therefore it is unreasonable to compare results for the plates with large perforation ratios. Second, the squeeze-film damping can be modeled by two approaches using ANSYS. In the first approach, the fluid in air gap is modeled by the Navier-Stokes equation, which is solved by using 3-D elements in FEM. In the second approach, the fluid is modeled by the Reynolds equation, which is solved by using 2-D elements (FLUID 136 and 138 elements). In fact, the first approach is the most accurate way to predict the squeeze-film damping. However, 3-D FEM simulation for the squeeze-film damping in the perforated plate is time consuming and non-transparent. In contrast, the second approach is simple and highly efficient. The FEM results in this paper are obtained by using the second approach. However, the second approach has a limitation. Only the damping force acting on the lower surface of the perforated plate is considered in the second approach. In the case of large perforation ratios, the area of the lower surface of the perforated plate is very small. When the perforation ratio approaches 100%, the area of the lower surface approaches zero, so the damping force obtained by the second approach is zero. The damping force caused by the mechanical resistance of the channel is not considered in the second approach. The damping force caused by the mechanical resistance of the channel is not zero in the case of large perforation ratios. Thus total damping force is not zero in the case of large perforation ratios. The damping force caused by the mechanical resistance of the channel is considered in the present model. A detailed discussion for the limitation of the second approach can be found in [2].

To examine the validity of the present model over a wide range of frequency, we compare the damping torques and the spring torques obtained by the present model and the FEM model in the range from 1 kHz to 1000 MHz. It needs to be stressed that the derivation in this paper assumes the modified Reynolds number to be small (Re=ρairωg02μ<<1) and neglects inertial effects. This sets an upper limit for the torsion devices on the maximum frequency. Above the maximum frequency, the inertia terms cannot be neglected. It is unreasonable to compare the results above the maximum frequency. However, the inertia effect is also not included in the FLUID136 element. Thus it is reasonable to compare the results at arbitrary frequency.

**Figure 8:**
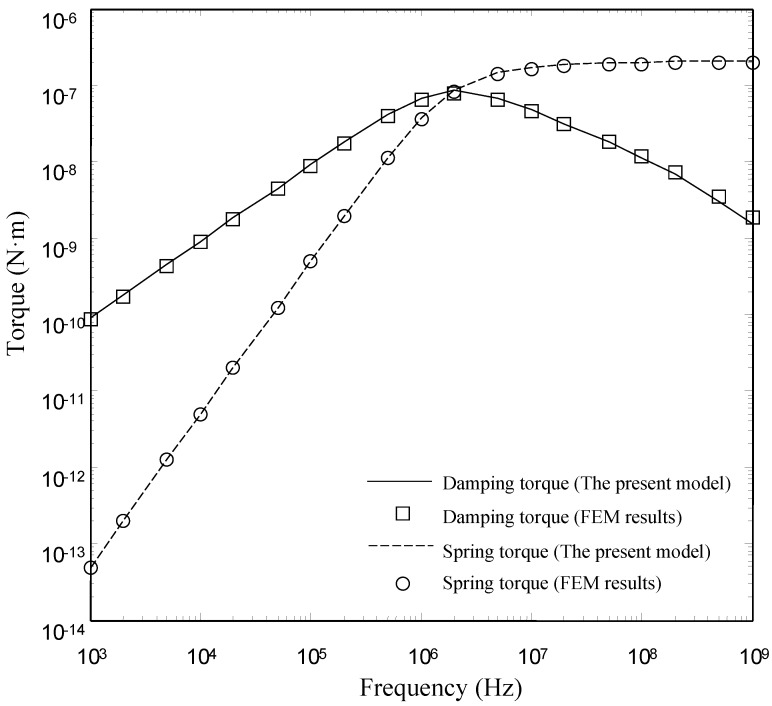
Comparison of the damping torques and the spring torques for microplate (*l_h_*/*l_p_* = 0.2) obtained by the FEM model and the present model as a function of frequency.

**Figure 9:**
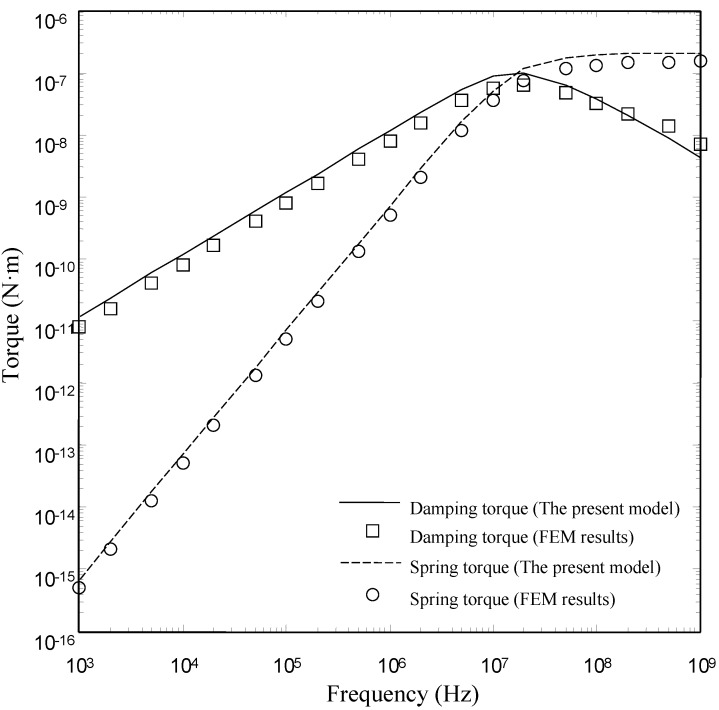
Comparison of the damping torques and the spring torques for microplate (*l_h_*/*l_p_* = 0.5) obtained by the FEM model and the present model as a function of frequency.

**Figure 10:**
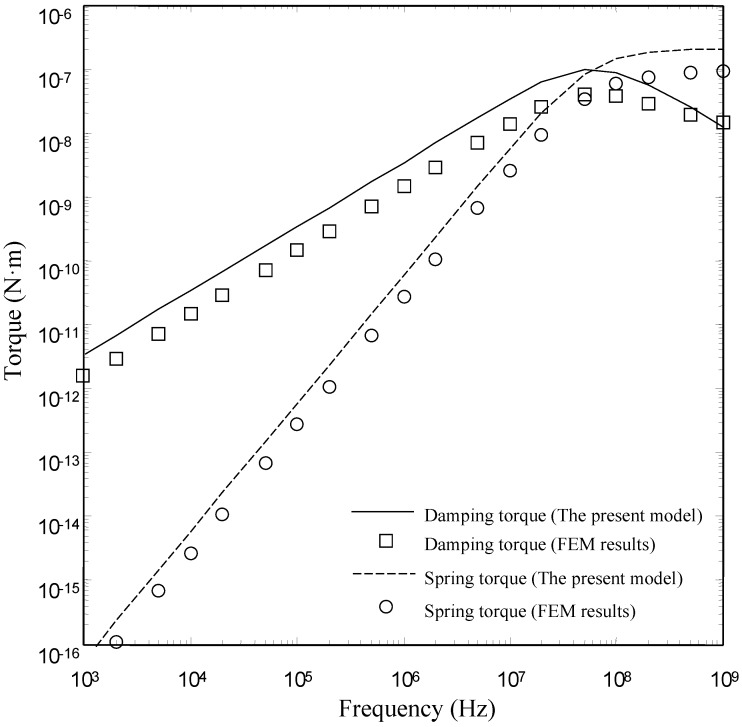
Comparison of the damping torques and the spring torques for microplate (*l_h_*/*l_p_* = 0.7) obtained by the FEM model and the present model as a function of frequency.

Figure 8, Figure 9 and Figure 10 show the damping torques and the spring torques obtained by the two models as a function of frequency for the microplates with *l_h_*/*l_p_* = 0.2, 0.5 and 0.7 respectively. As expected, the present model matches well with the FEM results over a wide range of frequency for the microplates with *l_h_*/*l_p_* = 0.2 and 0.5. The discrepancy between the present model and the FEM model also increases as the perforation ratio increases. There is a clear discrepancy between the FEM model and the present model at large perforation ratios.

### 4.2. Comparsions with the FEM Results for the Type II Torsion Microplate

**Figure 11:**
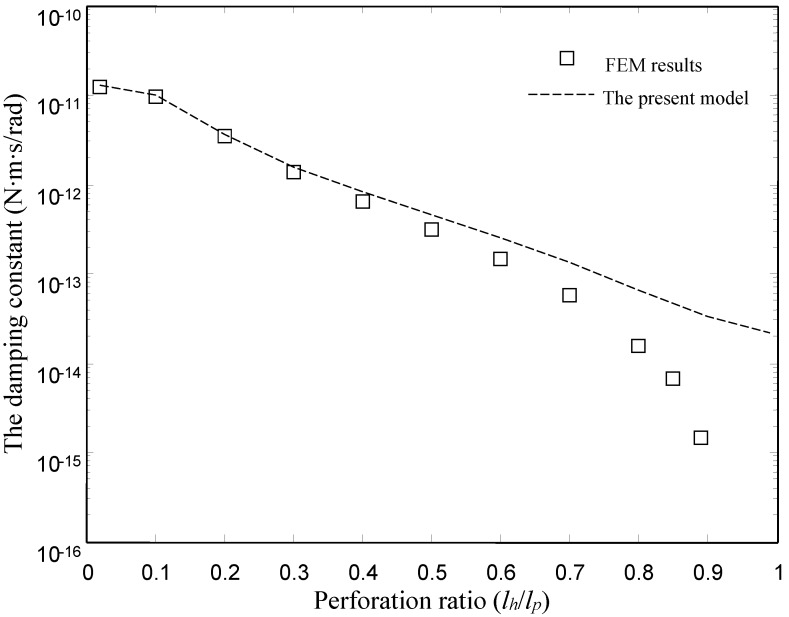
Comparison of the damping constant of the type II devices obtained by the FEM model and the present model.

**Figure 12:**
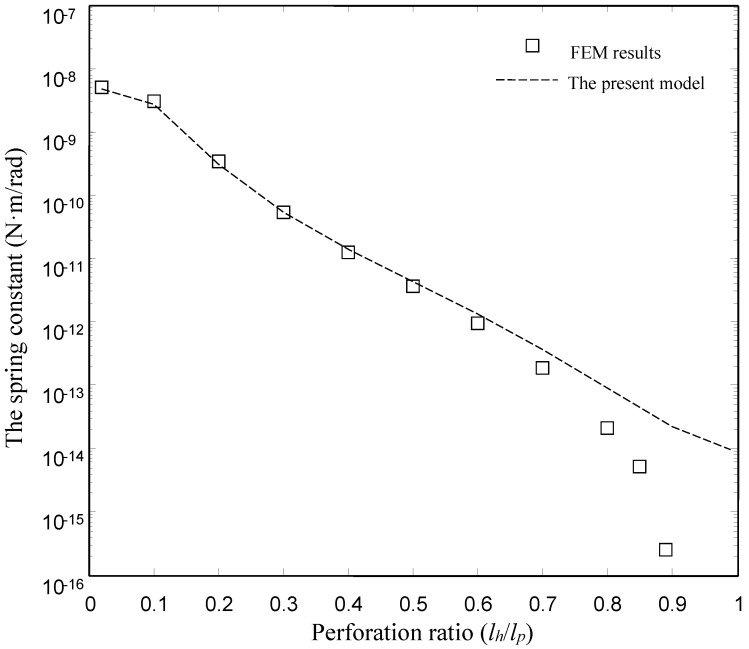
Comparison of the spring constant of the type II devices obtained by the FEM model and the present model.

The torsion microplate with 50 holes (*N_x_* = 5 and *N_y_* = 10) shown in Figure 3 is considered in this subsection. The torsion plate is asymmetric with respect to the rotation axis. The length of the torsion microplate is *L_x_* = 250 µm and other dimensions are as mentioned in Table 3. Figure 11 and Figure 12 show the damping constants and spring constants of the microplates as a function of perforation ratio (*l_h_*/*l_p_*) obtained by the FEM model. Also shown in the same figures are the analytical results obtained by the present model. Table 5 lists the detailed values of the damping and spring constants obtained by the ANSYS model and the present model. In ANSYS, the torsion microplate is operated at 5000 Hz. The amplitude of the vibrating plate is very small ($A_{0}=\frac{g_{0}}{10L_{x}/2}$). As expected, the present model matches well with the FEM results for the plates with the smaller and medium perforation ratios. For the damping constants, in the case of smaller and medium perforation ratios (*l_h_*/*l_p_* ≤ 0.6), the present model gives values very close to the FEM results. For the spring constants, below *l_h_*/*l_p_* = 0.7, the present model gives values very close to the FEM results. There is a clear discrepancy between the FEM model and the present model at large perforation ratios.

To check the validity of the present model at different values of frequency, we also compare the damping torques and the spring torques obtained by the present model and the FEM model in the range from 1 kHz to 1000 MHz. The tendency of the results for the type II plates are similar to that for the type I plates, *i.e.*, the present model matches well with the FEM results over a wide range of frequency for the plates with the smaller and medium perforation ratios. For the purpose of brevity, the figures for the type II microplates at different values of frequency are not shown in this subsection.

**Table 5:** The damping and spring constants obtained by the ANSYS model and the presnt model for the torsion microplate (Type II).

$\frac{l_{h}}{l_{p}}$	The Damping Constant		The Spring Constant	
	FEM Model	The Present Model	FEM Model	The Present Model
0.02	1.26 × 10^−11^	1.26 × 10^−11^	4.63 × 10^−9^	4.67 × 10^−9^
0.1	9.71 × 10^−12^	9.84 × 10^−12^	2.69 × 10^−9^	2.73 × 10^−9^
0.2	3.47 × 10^−12^	3.58 × 10^−12^	3.10 × 10^−10^	3.10 × 10^−10^
0.3	1.37 × 10^−12^	1.55 × 10^−12^	4.82 × 10^−11^	5.34 × 10^−11^
0.4	6.42 × 10^−13^	8.18 × 10^−13^	1.15 × 10^−11^	1.43 × 10^−11^
0.5	3.19 × 10^−13^	4.59 × 10^−13^	3.24 × 10^−12^	4.40 × 10^−12^
0.6	1.48 × 10^−13^	2.54 × 10^−13^	8.39 ×10^−1^^3^	1.32 × 10^−12^
0.7	5.83 × 10^−14^	1.33 × 10^−13^	1.70 × 10^−13^	3.60 × 10^−13^
0.8	1.56 × 10^−14^	6.62 × 10^−14^	1.93 × 10^−14^	8.80 × 10^−14^
0.85	6.66 × 10^−15^	4.99 × 10^−14^	4.64 × 10^−15^	5.55 × 10^−14^
0.9	1.46 × 10^−15^	3.37 × 10^−14^	2.29 × 10^−16^	2.28 × 10^−14^
0.99	-	2.19 × 10^−14^	-	9.59 × 10^−15^

### 4.3. Comparsions with the Experimental Results of Pandey et al. [21]

Pandey *et al.* [21] conducted experiments to measure the quality factors of a double-gimballed torsion micro-resonator in the free molecular regime. In the dual axis resonator, there are two types of motion, one about the inner torsional axis and the second about the outer torsional axis. Pandey *et al.* [21] measured the quality factors of the two motions as a function of pressure in the range from 10^−3^ to 10^3^ Torr. In this subsection, only the experimental data of the motion about the inner torsional axis are used to examine the presented model. The main reason is as follows: for the motion about the inner torsional axis, only the inner plate vibrates with angular displacement and the rest of the structure is stationary. The inner plate is a rectangular perforated microplate. The inner plate is asymmetry with respect to the rotation axis. The plate has the following dimensions: *L_x_* = *L_y_* = 400 μm, *N_x_* = *N_y_* = 12, *l_h_* = 3 μm, *l_p_* = 32.5 μm *T_p_* = 4.25 μm and $g_{0}$ = 80 μm. The plate is operating at its natural frequency ɷ = 529.2 Hz. Air is used as fluid medium. The temperature is *T* = 293 K. The moment of inertia about the inner axis is $\hat{J}$ = 2.106 × 10^−17^ kg·m^2^.

The basic assumption in Reynolds equation is *g*_0_*/L_x_* << 1. The flow in the *z*-direction can be negligible. However, for the inner plate of Pandey *et al.* [21], the gap spacing is comparable to the lateral dimensions (*g_0_/L_x_* = 0.2). To model the squeeze-film damping under such conditions using the models based on the Reynolds equation, Pandey and Pratap [22] replaced the length and width of the vibrating plate with the effective length and width. The effective length and width are computed by comparing the analytical results based on the Reynolds equation to the numerical results obtained by 3-D FEM. The effective length and width presented by Pandey and Pratap [22] are $L_{x}^{\text{eff}}=L_{x}+1.425g_{0}$ and $L_{y}^{\text{eff}}=L_{y}+1.425g_{0}$. Here, we use the present model with the effective length and width to predict the quality factors of the inner plate.

**Figure 13:**
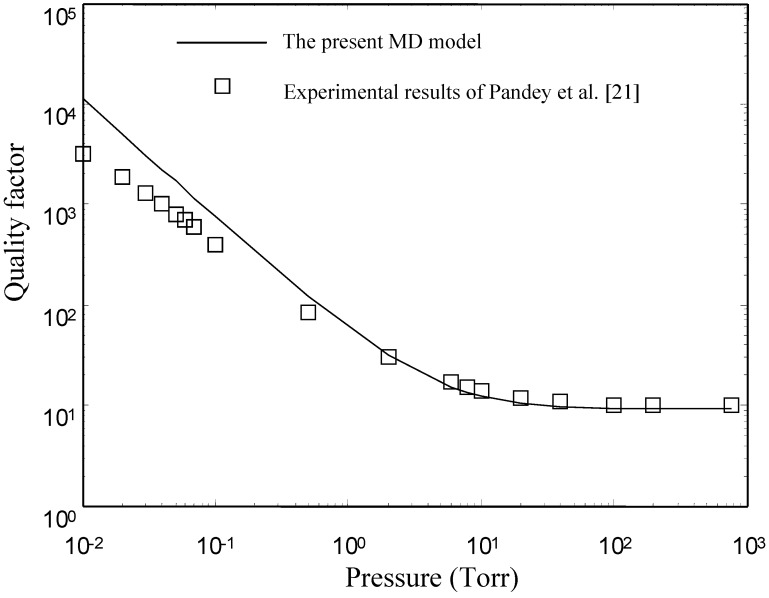
Comparison of the quality factors obtained by the present model to the experimental results of Pandey *et al.* [21].

Figure 13 shows the experimental results obtained by Pandey *et al.* [21]. Also shown in the same figure are the results obtained by the present model with the effective length and width. Obviously, above *p* = 0.5 Torr, the present model matches well with the experimental results. However, there is a clear discrepancy between the present model and the experimental results below *p* = 0.5 Torr. Two reasons for the discrepancy are as follows: first the present model treats the gas in the gap as a continuum. However, below *p* = 0.5 Torr, *K*n > 1, the flow is in the transition region or in the free molecular region. The continuum theory may fail to give a good prediction in the two regions. The second reason for the discrepancy is the intrinsic damping of the material. There are two mechanisms of energy dissipation in the experimental results. One is the air damping. The other is the intrinsic damping of the material. The damping obtained by the experimental results including the two damping can be expressed by:

(33) $$\frac{1}{Q_{\text{Exp}}^{\text{All}}}=\frac{1}{Q_{\text{Exp}}^{\text{squeeze}}}+\frac{1}{Q_{\text{Exp}}^{\text{intrinsic}}}$$

where $Q_{\text{Exp}}^{\text{All}}$ is the experimental result obtained by Pandey *et al.* [21], ${\left(Q_{\text{Exp}}^{\text{squeeze}}\right)}^{-1}$ is the squeeze-film damping of the air gap and $Q_{\text{Exp}}^{\text{intrinsic}}$ is the intrinsic damping. In low pressure, $Q_{\text{Exp}}^{\text{squeeze}}$ is proportional to air pressure. However, in very low pressure, the intrinsic damping begins to dominate the energy loss. The intrinsic damping is independent of the air pressure.

## 5. Conclusions

Perforations in microstructures play a significant role in controlling the squeeze-film damping. However, there are few works on analytical modeling of the squeeze-film damping of torsion perforated microplates. There are two types of torsion perforated plates used in micro-resonators. This paper presents two analytical models for calculating the squeeze-film damping in both types of torsion plates. The pressure in the air gap is obtained using the double sine series. Closed-form expressions for the stiffness and damping coefficients of the squeeze-film are derived. On comparing the present model with the FEM results and experimental results available in the literature, the following conclusions can be drawn.

(1) The present model is valid for devices with smaller and medium perforation ratios. The present model gives good results for the devices with *l_h_*/*l_p_* ≤ 0.6. For the devices with *l_h_*/*l_p_* ≤ 0.6, the present model matches well with the numerical model over a wide range of frequency. However, there is a clear discrepancy between the FEM model and the present model at large perforation ratios.
(2) The main assumption in this paper is the negligence of the inertial effect. The assumption limits the operating frequency range. Currently, we are extending this work to account for the inertial effect.
